# Gene expression profiling for the diagnosis of acute leukaemia

**DOI:** 10.1038/sj.bjc.6603495

**Published:** 2006-12-05

**Authors:** T Haferlach, A Kohlmann, U Bacher, S Schnittger, C Haferlach, W Kern

**Affiliations:** 1 MLL Munich Leukemia Laboratory, Max-Lebsche-Platz 31, Munich 81377, Germany; 2 Roche Molecular Systems, Inc., Pleasanton, CA, USA; 3 Department of Clinical Chemistry, Ludwig-Maximilians-University of Munich, Munich 81377, Germany

**Keywords:** microarray analysis, gene expression profiling, acute leukaemia, diagnosis

## Abstract

An optimised diagnostic setting in acute leukaemias combines cytomorphology and cytochemistry, multiparameter immunophenotyping, cytogenetics, fluorescence *in situ* hybridisation, and polymerase chain reaction (PCR)-based assays. This allows classification and definition of biologically defined and prognostically relevant subtypes, and allows directed treatment in some subentities. Over the last years the microarray technology has helped to quantify simultaneously the expression status of ten thousands of genes in single experiments. This novel approach will hopefully become an essential tool for the molecular classification of acute leukaemias in the near future. It can be anticipated that new biologically defined and clinically relevant subtypes of leukaemia will be identified based on their unique gene expression profiles. This method may therefore guide therapeutic decisions and should be investigated in a diagnostic setting in parallel to established standard methods.

An optimised routine diagnostic setting in acute leukaemias comprises a combination of methods including cytomorphology, multiparameter immunophenotyping, cytogenetics, fluorescence *in situ* hybridisation (FISH), and molecular methods such as polymerase chain reaction (PCR). This widened diagnostic spectrum has revealed deeper insights into disease-specific chromosomal and molecular alterations in acute leukaemias and has also led to improved understanding of the genetic heterogeneity of the diverse subtypes. Algorithms for diagnostic questions using these methods in varying combinations are helpful to gather all relevant information in an effective way (Haferlach *et al*, 2005a) and allow the selection of disease-specific therapeutic approaches, for example, use of all-*trans* retinoic acid in acute promyelocytic leukaemia (APL) with PML-RARA, of imatinib in BCR-ABL-positive acute lymphoblastic leukaemia (ALL), or of specific antibodies against the CD20 or CD52 antigens. Nevertheless, well-defined cytogenetic subgroups exhibit considerable heterogeneity with respect to both response to therapy and prognosis (Moos *et al*, 2002). Thus, more diversified classification systems based on the underlying molecular pathogenic event would be desirable (Bullinger and Valk, 2005).

Microarray technology provides comprehensive data on expression patterns of ten thousands of genes in parallel, which qualifies this method for a central role in the optimisation of diagnosis and of the classification of acute leukaemias. Gene expression profiling (GEP) may lead to the detection of new biologically defined and clinically relevant subtypes in leukaemias as a basis for specific therapeutic decisions (Valk *et al*, 2004). Testing as a routine method for diagnostic purposes in parallel to current standard methods is essential to enable GEP to be included in future routine diagnostic applications and in clinical trials (Haferlach *et al*, 2005c).

## TECHNICAL ASPECTS OF MICROARRAY ANALYSES

Microarrays contain precisely positioned DNA-probes designed to specifically monitor the expression levels of thousands of genes in a parallel manner. Commercial chip designs, for example, HG-U133 Plus 2.0 (Affymetrix, Santa Clara, California, USA), contain about 42 000 genes probably representing most of the human genome. Common to all expression profiling approaches is the heteroduplex formation: structural features of nucleic acid enable every nucleic acid strand to recognise complementary sequences through base pairing. After hybridisation, complementary fluorescently tagged nucleotides can be detected (Lockhart *et al*, 1996; Southern *et al*, 1999).

Different microarray platforms are available: filter arrays (formerly considered as macroarrays owing to their lower probe density), spotted glass slide arrays that vary according to the immobilised probe, that is, cDNA, oligonucleotides, or genomic fragments, and according to substrate choice for surface modification. With cDNA arrays, PCR products of cDNA clone representing genes of interest are spotted systematically on nitrocellulose filters or glass slides. The construction of spotted arrays is based on the use of cDNA collections that can be focused on genes expressed in a particular context. This array technique can be performed by individual investigators, is easily customisable, and does not require primary knowledge of cDNA sequence because clones can be used before sequencing. Oligonucleotide arrays offer greater specificity than cDNA arrays, because they can be tailored to minimise chances of cross-hybridisation (Ramaswamy *et al*, 2001).

Kohlmann *et al* (2005) demonstrated that preparation by different operators and use of different sample-handling procedures did not impair the robustness of diagnostic gene expression signatures. These results give further support to the application of microarrays in a routine diagnostic setting (Kohlmann *et al*, 2005).

The advanced computational methods employed in array-based classification – for example, support-vector machines – must be calibrated with large sets of example gene expression profiles. Cross-platform approaches, meaning generation of the training-set on a remote microarray set-up, different from the one locally used for analysing new samples, were demonstrated to be more consistent and reproducible than previously assumed (Nilsson *et al*, 2006), which further qualifies GEP to play a key role in routine diagnostic settings.

## DATA MINING, INTERPRETATION, AND STORAGE OF MICROARRAY DATA

The enormous amount of data generated by microarray analyses is a challenge even in small studies, easily summing up to several gigabyte of raw expression data. After image processing, the first analysis step is to produce a large number of quantified gene expression values. Normalisation must be performed before analysing the data in order to guarantee the appropriate comparison of the measured gene expression levels between a set of arrays.

Data mining, which is the discovery of non-obvious information, mainly uses two different analyses: supervised analyses group the patients according to predefined characteristics and thus allow a correlation of array data with already known parameters such as clinical parameters or outcome. Unsupervised analyses are used to test the hypothesis whether specific characteristics, for example, genetic aberrations, are also reflected at the level of gene expression signatures without predefining groups of interest.

A multiclass statistical analysis approach is highly recommended for acute leukaemias, as often multiple groups of leukaemia subtypes have to be compared. Variants of common statistical tests select gene expression levels that allow the separation of these known groups. After detecting differential gene expression, many different machine-learning algorithms are applied to accurately classify samples into these known groups. Ideally, class prediction is done by dividing gene expression data into training and independent test sets.

Hierarchical clustering allows the organisation of data into groups with similar signatures. It can be used for both reduction of complexity of the matrix-like data and visualisation in a more understandable way and predicts the categorisation of unknown samples. This hierarchical structure provides potentially useful information about the relationship between adjacent clusters. Common crossing points in dendrograms represent similar patient characteristics as well as similarities with regard to expression of distinct genes in gene expression patterns. Thus, it not only can be used for the visualisation of supervised analyses, but also is the method-of-choice, when information with respect to the complete repertoire of expected gene expression patterns is limited.

Principal component analysis (PCA) can be used to reduce the dimensionality of array data and to visualise large data sets. The multidimensional and matrix-like structured array data set is reduced to a new set of variables, that is, principle components.

To allow the direct estimation of biologic relevance, an effective annotation of microarray experiments is a major task that has been approached by the MGED group (Microarray Gene Expression Data Group, www.mged.org), which defined the standards for annotation and publication of microarray experiments.

## DIAGNOSIS OF ACUTE LEUKAEMIAS BASED ON GEP

The pivotal work of GEP in acute leukaemias was reported by Golub *et al* (1999) who provided data on the applicability of microarrays and new biostatistical analyses which led to a first ‘class prediction’: a limited pattern of 50 discriminatory genes was able to separate 27 patients with ALL from 11 patients with AML. Acute lymphoblastic leukaemia was further separated in T-lineage and in B-lineage. Subsequently, in 36 out of the 38 cases the molecular diagnosis of leukaemia was made correctly based on this gene expression profile (Golub *et al*, 1999; Ramaswamy *et al*, 2001). A separate study confirmed this discrimination of AML and ALL in 51 childhood leukaemias, and subdivided B-precursor- from T-precursor-ALL (Moos *et al*, 2002). An important milestone in microarray analysis with respect to class discovery and prediction of class and outcome was the report on childhood ALL by Yeoh *et al* (2002), who performed GEP subclassification according to the cytogenetic, immunological, and molecular subtypes, and were able to predict therapeutic outcome and to show that specific genes in ALL at diagnosis appear to indicate an increased risk of developing therapy-induced AML. Thus, the prediction of response to therapy, of risk to relapse, and even of risk of the development therapy-induced secondary malignancies seem realistic goals for GEP.

## AML

Recent studies were able to demonstrate that GEP shows significant correlations with all the established diagnostic methods applied so far in the acute leukaemis. As will be shown in further detail, there are strong correlations with cytomorpholgy, immunphenotyping, cytogenetics, and molecular genetics.

## MORPHOLOGY IN AML

The morphologic classification on the basis of the FAB and World Health Organization (WHO) classifications still plays an important role in acute leukaemia diagnostics owing to its fast and easy applicability, and the possibility of guidance of other diagnostic methods. Haferlach *et al* (2002) demonstrated that the prediction of the FAB subtype of AML is possible, based solely on the expression status of a limited set of one to three genes, which were sufficient to separate M3, M3v, M4eo, and M6 from all other subtypes with 100% accuracy. The prediction of morphology in APL subtypes, M3 and M3 variant, was also accomplished, although both show the t(15;17)/*PML-RARA*. This further confirms the diversity of both morphologic subtypes not only on the basis of morphologic aspects but also on the clinical outcome that seems better in the M3 subtype than in the M3v subtype (Haferlach *et al*, 2005b).

## IMMUNOPHENOTYPING IN AML

Until now, immunophenotyping by multiparameter flow cytometry has been the standard method for diagnosis and classification of acute leukaemias, especially ALL. In genes most relevant for diagnosis and subclassification of AML and ALL, Kern *et al* (2003) found congruent results between protein expression and corresponding mRNA abundance in 75–100% of all the 39 analysed AML cases. Thus, GEP correlates to protein expression data revealed by multiparameter flow cytometry.

## CYTOGENETICS IN AML

Cytogenetic aberrations are considered disease-defining in a large number of AML types. They represent the most important prognostic parameter, and have thus been incorporated into the WHO classification of AML (Jaffe *et al*, 2001). Identification is performed by chromosome banding analysis in combination with FISH and RT–PCR.

The reciprocal translocations t(8;21)/*AML1-ETO*, t(15;17)/*PML-RARA*, and inv(16)/*CBFB-MYH*11 are characterised by distinct morphologic phenotypes and by a favourable prognosis; reciprocal translocations involving the *MLL* gene on 11q23 are, by contrast, associated with poor prognosis and are frequent in therapy-related AML (t-AML). These reciprocal translocations represent the first hierarchy in the WHO classification of AML. Several groups were able to characterise different and specific gene expression profiles of these subgroups in adults (Schoch *et al*, 2002a, 2002b; Debernardi *et al*, 2003; Valk *et al*, 2004) and in children with AML (Ross *et al*, 2003). In an analysis of Kohlmann *et al* (2001), the expression profiles of 35 genes were sufficient for predicting the class of all samples of the above-mentioned three cytogenetic subtypes and for separating them from normal bone marrow with 100% accuracy. The application of a minimum set of 39 genes allowed the definition of a fifth AML subgroup, being represented by the 11q23/*MLL* gene rearrangements (Kohlmann *et al*, 2003). Ross *et al* (2003) confirmed the high prediction accuracy of 98% for AML with t(11q23)/*MLL* rearrangements in childhood AML. In addition, the authors could delineate APL with t(15;17)/*PML-RARA*, core-binding factor (*CBF*)-leukaemias, and acute megakaryocytic leukaemia with a 100% prediction accuracy in 130 children with AML. In conclusion, GEP confirms the status of these cytogenetic subtypes as distinct biologic entities provided in the WHO classification.

Trisomy 8 represents a frequent karyotype abnormality in AML as well as isolated change as in combination with other abnormalities. With respect to this chromosomal abnormality in AML, a complete separation with GEP from AML with normal karyotype was not possible in the study of Virtaneva *et al* (2001), who performed GEP on CD34+ cells on first-generation oligonucleotide arrays (Affymetrix). This probably reflects that trisomy 8 does not represent a disease-defining abnormality, but occurs somewhat as a secondary aberration. Nonetheless, a gene dosage effect was clearly demonstrated as many genes coded on chromosome 8 were expressed at higher levels in AML with trisomy 8.

Because of its very poor clinical course and its characteristic, but not yet well understood biologic features, AML with a complex aberrant karyotype (defined by ⩾3 chromosomal abnormalities) is an outstanding subtype. Schoch *et al* (2002a, 2002b) compared this subtype to AML with the reciprocal translocations t(8;21)/*AML*1-*ETO*, inv(16)/*CBFB-MYH*11, *MLL* rearrangements, trisomy 8 as sole abnormality, and normal karyotype in 150 cases (Schoch *et al*, 2002a, 2002b). The discrimination of AML with complex aberrant karyotype from every other subgroup was possible with 100% accuracy in pairwise comparison. In addition there was a significantly higher expression of *RAD*21 (1.7-fold), which is involved in double-strand break repair and has antiapoptotic funtion in AML. This confirms the separation of this AML subtype from other cytogenetic subgroups.

## MOLECULAR GENETICS IN AML

Forty-five per cent of all AML patients show a normal karyotype and represent the largest subgroup. In recent years, the spectrum of recurrent molecular mutations in AML has considerably broadened. Around 75–80% cases of normal karyotype can now be further classified by molecular methods. For example, the heterogeneous mutations affect receptor tyrosine III kinases such as the *FLT*3 kinase, partial tandem duplications within the *MLL* gene with its many funtions in haematopoiesis, and *NPM*1 mutations, which affect a nucleocytoplasmic shuttle protein with involvement in a tumour-suppressor-pathway. These molecular markers are not randomly distributed, but are associated with distinct cytogenetic subgroups and represent for some part independent prognostic parameters.

The *FLT*3 length mutations (*FLT*3-LM) (or *FLT*3-ITD; internal tandem duplication) represent a frequent molecular mutation in AML found in 23% of all cases and in 40% of all normal karyotype, and are highly associated with a negative prognosis. Schnittger *et al* (2002a) compared the expression profile of AML with *FLT*3-LM to normal bone marrow samples, AML with t(8;21)/*AML*1-*ETO*, inv(16)/*CBFB-MYH*11, t(15;17)/*PML-RARA*, *MLL*-translocations, trisomy 8, and complex aberrant karyotype. The *FLT*3-LM group was discriminated from trisomy 8 cases with 97% accuracy and from all other karyotypically aberrant AML groups with 100% accuracy. However, it was not possible to discriminate within AML with normal karyotype between those with and without *FLT*3-LM. Neither was it possible after including point mutations in the tyrosine kinase domain (*FLT*3-TKD) cases into the *FLT*3 mutated group. Within the distinct cytomorphologic FAB subgroups, however, a clear separation between *FLT*3-LM positive and negative cases was accomplished. The 20 top discriminative genes varied substantially between the diverse FAB subtypes, although many are downstream target genes of *FLT*3. These data suggest that the effects of a mutationally activated *FLT*3 receptor may be different, depending on a primary genetic alteration or the composition of different genetic alterations in addition to the *FLT*3-LM. These additional alterations may vary between the distinct morphological subtypes, and thus cause a differentiation block at different levels in haematopoiesis.

In a series of 110 AML patients with normal karyotype, Neben *et al* (2005) were able to separate samples with *FLT*3-LM and *FLT*3-TKD, with up to 100% accuracy. This did not apply to *NRAS* mutations and *NRAS* wild-type samples, suggesting that only *FLT*3-LM and *FLT*3-TKD are associated with a specific signature (Neben *et al*, 2005). In a similar approach, Lacayo *et al* (2004) were able to identify cases with *FLT*3-LM, *FLT*3-TKD, and those without either mutation in a series of 81 childhood AML, although there were significant overlaps between the respective groups (Lacayo *et al*, 2004).

Partial tandem duplications of the *MLL* gene (*MLL-*PTD) occur mainly in cytogenetically normal AML and are prognostically unfavourable. It was not possible to define a specific expression profile discriminating positive from negative cases (Schnittger *et al*, 2002b). By contrast, Ross *et al* (2003) showed that *MLL* chimeric fusion genes are characterised by a distinct expression signature in childhood acute leukaemia irrespective of lineage assignment. AML with *MLL*-PTD did not cluster with *MLL* chimeric fusion gene cases (Ross *et al*, 2003). Thus, the pathogenic mechanisms of partial duplications and of chimeric gene fusions of the *MLL* gene seem to differ significantly. Further, these results suggest that the *MLL*-PTD might represent an example of a mutation that does not define a specific distinct biologic entity but instead is involved in different subtypes of leukaemias.

*NPM*1 mutations are also correlated with normal karyotype in AML and predict favourable survival if detected as the only molecular alteration. The predictability of *NPM*1 mutations on the basis of GEP results is controversial: Verhaak *et al* (2005) did not succeed in showing, in an unsupervised analysis, a clearcut separation of *NPM*1 mutated from unmutated cases in more than 100 AML patients with normal karyotype. By contrast, the unsupervised clustering analyses of Alcalay *et al* (2005) clearly separated *NPM*1 mutated from *NPM*1 wild type regardless of the presence of additional *FLT3* mutations or nonmajor chromosomal rearrangements. This is strongly supporting *NPM*1 mutations in AML as a distinct biological entity (Alcalay *et al*, 2005). The molecular signature of *NPM*1 mutated AML includes upregulation of several genes putatively involved in the maintenance of a stem-cell phenotype. Similarly, Wilson *et al* (2006) found *NPM*1 mutations highly correlated with a cluster that was characterised by normal karyotype, genes involved in signalling and apoptosis, and an excellent prognosis in a study on 170 AML patients. In addition, *NPM*1 mutations were associated with a cluster of monocytic leukaemias.

Valk *et al* (2004) and Bullinger and Valk (2005) focused on AML with normal karyotype with respect to other molecular markers and prognosis. Valk *et al* (2004) used unsupervised cluster analyses and identified up to 16 groups of AML based on separate molecular signatures. The clustering was driven not only by the presence of chromosomal lesions (e.g., t(8;21)/*AML1-ETO*, t(15;17)/*PML-RARA*, inv(16)/*CBFB-MYH11*), but also by particular genetic mutations (*CEBPA*, *MLL*-PTD, *FLT*3-LM) and abnormal oncogene expression (*EVI*1). Thus, GEP seems suitable for the characterization of the large subgroup of AML with normal karyotype. Bullinger was able to separate distinct subgroups with different prognosis within AML and normal karyotype on specific GEPs.

## ACUTE LYMPHOBLASTIC LEUKAEMIA

Following the results of Golub *et al* (1999) who had shown that the definition of the lineage of acute leukaemias can be predicted on the basis of a small number of genes, further subclassification of ALL on the basis of GEP was performed showing that most cytogenetic, molecular, and immunologic subgroups of ALL can be linked to distinct gene signatures.

## CYTOGENETICS AND MOLECULAR GENETICS IN ALL

Moos *et al* (2002) divided childhood leukaemia samples into AML, B-lineage ALL, and T-lineage ALL, and were able to separate low-risk ALL from high-risk ALL cases. They were able to demonstrate that the *TEL-AML*1/t(12;21) fusion transcript with its high frequency in childhood ALL and its favourable prognosis represents a distinct gene signature in ALL (Moos *et al*, 2002).

*MLL*/11q23 rearrangements define a prognostically unfavourable subgroup in ALL. In the study of Armstrong *et al* (2002), Armstrong *et al MLL*-positive ALL had a distinct GEP consistent with an early haematopoietic progenitor cell expressing multilineage markers and specific *HOX* genes. This entity could be clearly separated from other ALL and AML cases (Armstrong *et al*, 2002).

In the above-mentioned study, Yeoh *et al* (2002) discriminated 327 childhood ALL cases in the following subtypes: T-ALL, *E*2*A-PBX*1/t(i;19), *BCR-ABL*/t(9;22), *TEL-AML*1/t(12;21), *MLL*/11q23 rearrangements, and hyperdiploid karyotype. Kohlmann *et al* (2003) could accurately discriminate in adult ALL precursor B-ALL with t(9;22)/*BCR-ABL* and t(4;11)/*MLL-AF4*, B-ALL with t(8;14)/*IgH-c-MYC*, respectively, and precursor T-ALL.

In contrast to most genetic ALL subtypes that could be associated to distinct gene signatures, the *BCR-ABL* gene expression pattern in Philadelphia-positive ALL was identified as more heterogeneous, similar to ALL without known molecular rearrangements in the study of Chiaretti *et al* (2005).

The major role of cytogenetic aberrations in the characterisation of acute leukaemia entities has further been proven in an analysis of ALL cases comparing the respective gene expression signatures in children and adults (Kohlmann *et al*, 2004). Using genes differentially expressed in childhood ALL subgroups, a classification of corresponding adult ALL cases resulted in high prediction accuracy compared with the study of Armstrong *et al* (2002). These analyses not only emphasise the importance of genetics in leukaemia across different age groups, but also prove the reproducibility of microarray analyses in general, leading to consistent data in separate analyses performed in different laboratories on independent samples.

## IMMUNOPHENOTYPING IN ALL

Kern *et al* (2003) analysed ALL cases combining immunologic and cytogenetic classifications – Pro-B-ALL/t(4;11), c-ALL/Pre-B-ALL with and without t(9;22), mature B-ALL/t(8;14), Pro-T-ALL, Pre-T-ALL, and cortical T-ALL. The prediction accuracy for discriminating T-precursor from B-precursor ALL was 100%. Although PCA of B-precursor ALL cases yielded distinct clusters for Pro-B-ALL, c-ALL/Pre-B-ALL, and mature B-ALL, the precursor subtypes c-ALL/Pre-B-ALL with t(9;22)/*BCR*-ABL were not completely discriminated from those without t(9;22)/*BCR-ABL*. This is in accordance with other analyses demonstrating the difficulty in identifying a specific gene expression signature for B-precursor ALL with t(9;22)/*BCR-ABL* (Yeoh *et al*, 2002; Chiaretti *et al*, 2005).

However, cortical T-ALL were found to show distinct clusters from immature T-ALL cases; yet, there was a large overlap between Pro-T-ALL and Pre-T-ALL and even biphenotypic acute leukaemias carrying both T-lymphatic and myeloid features (Kern *et al*, 2003). A further step towards better understanding of the biological characteristics of T-ALL has been suggested by Ferrando *et al* (2002) who identified distinct gene expression signatures and related them to normal thymocyte development (Ferrando *et al*, 2002; Ferrando and Look, 2003). Accordingly, Pro-T and *LYL*1-positive cases were distinguished from early cortical thymocyte and *HOX*11-positive cases as well as from *TAL*1-positive late cortical thymocyte cases.

## GLOBAL APPROACHES IN THE DIAGNOSIS OF LEUKAEMIAS USING MICROARRAYS

Several papers used GEP as a global approach for the diagnosis in acute leukaemias (Yeoh *et al*, 2002; Ross *et al*, 2003; Haferlach *et al*, 2005c). At present, an international study (MILE: microarray innovations in leukaemia) is prospectively testing 4000 cases of leukaemia and MDS in 11 centres within the European leukaemia network (ELN, WP13) to define the role of GEP in the diagnostic panel in leukaemia (Haferlach *et al*, 2006; Staal *et al*, 2006). Each centre is trained on an identical microarray protocol and uses the same equipment and kits for target preparation (Affymetrix HG-U133 Plus 2.0). For the first time, an international multicentre research study demonstrated a very high reproducibility of microarray analyses performed at different centres with a prediction accuracy was 95.6%. This lays the foundation for an international clinical research initiative evaluating the application of microarrays in the diagnosis and classification of haematologic malignancies (Haferlach *et al*, 2005).

In the past years, several studies further investigated the prediction of response to therapy and biologically directed class discovery using GEP in leukaemia. However, this review is intended to cover only the diagnostic possibilities of microarrays, so these important aspects and contributions cannot be demonstrated in detail.

## CONCLUSION AND FUTURE DIRECTIONS

The introduction of microarray technology has been a major step towards the comprehensive biologic characterisation of various diseases and will clearly allow the identification of yet unknown subentities and even new biologically defined entities. In particular, it has become clear that distinct cytogenetically defined subtypes in leukaemia have very specific underlying gene expression profiles that can be used to identify these subtypes based on microarray analyses with very high accuracy. The problems concerning the prediction of some less well-defined leukaemia subclasses might lead to the interpretation that these might not represent real subgroups but rather a heterogeneous mixture of different leukaemias; however, GEP might provide a tool to further delineate the respective subtypes.

It is expected that the routine application of microarrays will significantly improve molecular diagnostics in leukaemia (Staudt, 2003) and will provide new insights into the pathogenic alterations of malignant and non-malignant haematopoietic cells. In addition, these comprehensive data are anticipated to allow the identification of prognostically relevant markers and disease-specific markers that can be applied for collaborative programmes to monitor minimal residual disease (MRD). Of the highest clinical relevance is the capability of microarray approaches to identify pathogenically essential structures and alterations that can be targeted by future drugs that, hopefully, will lead to an improved management of these diseases.

The adequate diagnosis and subclassification of leukaemias today is based on a combination of various methods including cytomorphology, cytochemistry, multiparameter immunophenotyping, cytogenetics, fluorescence *in situ* hybridisation, and quantitative and nonquantitative molecular genetics. This is costly, time-consuming, and requires skilled personnel in centralised reference laboratories. Based on GEP, substantial steps forward have already been made in the direction of both optimising the diagnostic capabilities and reducing the financial reserves. A significant number of today's diagnostic approaches can already be reproduced by GEP, and further clinical trials are on the way to assert the validity of this approach for diagnosis, prognostication, and individual treatment decisions.
